# Estrogen Mediates Innate and Adaptive Immune Alterations to Influenza Infection in Pregnant Mice

**DOI:** 10.1371/journal.pone.0040502

**Published:** 2012-07-05

**Authors:** Michael A. Pazos, Thomas A. Kraus, César Muñoz-Fontela, Thomas M. Moran

**Affiliations:** 1 Department of Microbiology, Mount Sinai School of Medicine, New York, New York, United States of America; 2 Department of Obstetrics, Gynecology, and Reproductive Sciences, Mount Sinai School of Medicine, New York, New York, United States of America; 3 Heinrich-Pette-Institut, Leibniz-Institut für Experimentelle Virologie, Hamburg, Germany; University of California Los Angeles, United States of America

## Abstract

Pregnancy is a leading risk factor for severe complications during an influenza virus infection. Women infected during their second and third trimesters are at increased risk for severe cardiopulmonary complications, premature delivery, and death. Here, we establish a murine model of aerosolized influenza infection during pregnancy. We find significantly altered innate antiviral responses in pregnant mice, including decreased levels of IFN-β, IL-1α, and IFN-γ at early time points of infection. We also find reduced cytotoxic T cell activity and delayed viral clearance. We further demonstrate that pregnancy levels of the estrogen 17-β-estradiol are able to induce key anti-inflammatory phenotypes in immune responses to the virus independently of other hormones or pregnancy-related stressors. We conclude that elevated estrogen levels result in an attenuated anti-viral immune response, and that pregnancy-associated morbidities occur in the context of this anti-inflammatory phenotype.

## Introduction

The immunological implications of pregnancy have motivated immunologists for more than half a century [1]. Various mechanisms have been proposed to explain how rejection of an antigenically foreign fetus is prevented, from the absence of classical MHC class I molecules [2], to regulatory T cell expansion [3] and specific suppression of paternally-derived antigens [4], [5]. Pregnancy is also associated with reduced pathology and alleviation of symptoms in inflammatory autoimmune diseases such as multiple sclerosis [6] and rheumatoid arthritis [7]. The profound immunological changes in these disease states during pregnancy suggests that there exists a broad impact on immune regulation that is not specific to fetal antigens.

Empirical evidence suggests that pregnant women fair poorly in response to certain pathogen challenges, including *Listeria monocytogenes* [8], malaria [9], SARS coronavirus [10], [11], varicella zoster [12], rhinovirus [13] and influenza virus [14], among others. Of these, the increased risk of severe influenza infection is of particular public health concern. Pregnancy has been an acknowledged risk factor for severe complications from influenza virus infection for nearly a century [15], and has been observed during every major influenza pandemic including the pandemics of 1918 [15], 1957 [16], 1968 [17], and 2009 [18]–[20]. Implications of a severe influenza infection during pregnancy are life threatening for mother and child. Significantly increased risks of cardiopulmonary events have been reported [14], and mortality for the mother can be as high as 45% [21]. Moreover, consequences to the fetus range from behavioral alterations and low birthweight, to preterm birth and spontaneous abortion [21]–[24]. Significant effort has been put into determining the safety and efficacy of vaccination during pregnancy [25], [26], as well as the efficacy of neuraminidase inhibitors [20], [27], but a deeper understanding of the changes in maternal immunity during pregnancy are needed in order to devise adequate therapeutic strategies.

Many clinical studies have been performed to empirically define the increased severity of influenza infections during pregnancy [14], [18], [21]. For safety and ethical reasons, these studies are conducted in a setting without viral challenge, and rely on observations made only after the onset of symptoms. Although earlier work has established that the increased mortality to influenza infection during pregnancy can be observed in mouse models [28]–[31], recent work has primarily focused on characterization of associated morbidities in lethal infections with little insight into how immunity is impacted early in infection.

Increased morbidity due to influenza infection is typically associated with the third trimester of pregnancy, and correlates well with the highest levels of circulating estrogen. Estrogens have long been known to possess potent immunomodulatory effects in various models of disease [32]–[34], and have been proposed for therapeutic use in some clinical situations [35]. Although pregnancy levels of estrogen have been associated with reduced levels of immune-mediated morbidity, severe morbidity is a hallmark of influenza infection during pregnancy. We sought to reconcile these observations and determine what role high levels of estrogen play in influenza infections during pregnancy.

We have developed a model of influenza infection in pregnant mice and documented the major changes in the anti-viral response. Moreover, using implantable hormone pellets, we have determined that estrogen alone is able to recapitulate many key alterations observed in pregnancy. Estrogen treatment leads to early reductions in cytokine production, in particular type-I interferon (IFN), impacting downstream activation of adaptive immunity. Additionally, estrogen treatment compromises effective antigen-specific cytotoxicity. Ultimately these deficiencies result in significantly delayed viral clearance in pregnant animals, highlighting the presence of an anti-inflammatory mechanism in the context of enhanced morbidity.

## Results

### Altered Morbidity in Influenza-infected Pregnant Mice

**Figure 1:**
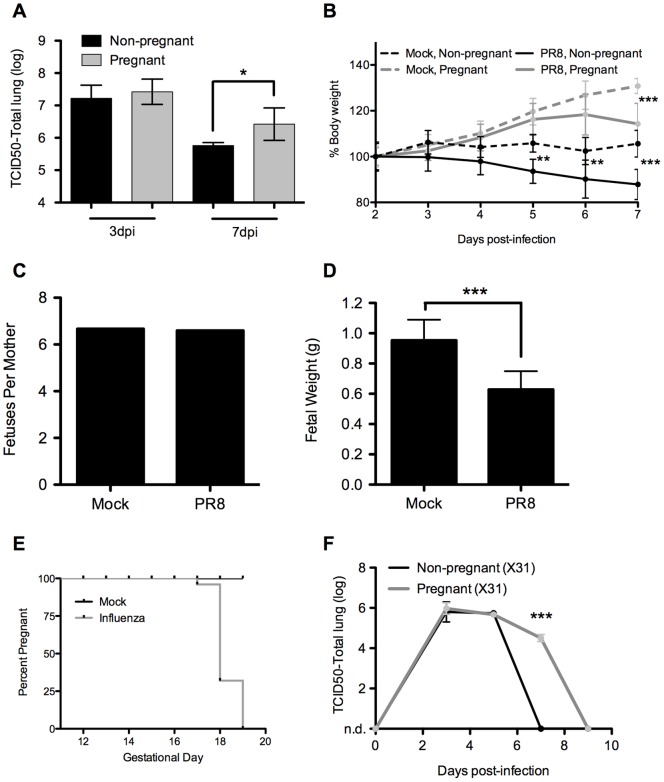
Altered influenza morbidity in pregnant mice. (A) Pregnant and non-pregnant C57BL/6 mice were infected with aerosolized PR8. At the indicated time points, mice were sacrificed and total lung titers were measured by tissue culture infectivity of MDCK cells (TCID50). (B) Weight changes in PR8-infected and mock-infected pregnant and non-pregnant mice were tracked through infection. (C) The average number of fetuses per mother was calculated at 8dpi, and (D) individual fetuses were weighed from mock infected or PR8-infected mothers. (E) Gestation of pregnant animals was tracked through the end of the experiment. All mock-infected pregnant mice remained pregnant until sacrifice. Data represents a compilation of multiple experiments including infections with X31 and PN-1. Results were consistent between virus strains. (F) Total lung titers were measured in pregnant and non-pregnant mice following an infection with aerosolized X31. Non-detectable levels are indicated as n.d. * p<0.05, ** p<0.01, ***p<0.001.

To determine the response to influenza virus infection during pregnancy, we challenged mice at gestational day 10.5 (E10.5) and non-pregnant controls with aerosolized PR8 virus in a controlled chamber. Peak viral titers were reached in both sets of mice by 3 day post-infection (dpi) (Figure 1A), as described previously [36]. By 7 dpi, non-pregnant mice showed significantly lower pulmonary viral titers, suggesting they were able to clear virus more efficiently than their pregnant counterparts.

Weight gain resulting from pregnancy outpaced any potential weight loss incurred during infection, although pregnant infected mice did lose weight compared to mock controls. This weight loss was delayed by approximately 2 days when compared to the weight loss of non-pregnant controls, suggesting that immune-associated morbidities were delayed in pregnant animals (Figure 1B). Significant morbidity was readily evident in measures of gestational health. The number of fetuses per mother were not effected by infection in our model (Figure 1C), but fetuses from influenza-infected mice failed to achieve the weight of their counterparts from mock-infected mothers. By E18.5 they were nearly 35% below the weight of controls (Figure 1D). The length of gestation was also significantly impacted by infection with influenza virus. Infected pregnant mice undergo labor much earlier than their mock-infected controls (Figure 1E). All mock infected pregnant mice continued gestation beyond the bounds of our experiments, while infected mice universally delivered by E19. Pups delivered by influenza-infected mothers were therefore not only premature, but additionally underweight for their gestational age. These results reinforce the evidence that the viability of the pregnancy is negatively impacted by infection with influenza virus, with a significant burden borne by the fetus, as is seen in clinical situations [23], [37].

As most clinical incidents of influenza infection are non-lethal, we wanted to model a viral infection with a milder pathology. PR8 is extremely virulent, and useful for establishing morbidities, but is 100% lethal in wild-type mice. As a non-lethal infection model, we made use of the reassortant mouse-adapted virus X31 (H3N2). Infection of pregnant animals with this virus leads to full recovery, but with a significantly more pronounced delay in viral clearance (Figure 1F). These data suggest that despite significant morbidity associated with fetal gestation, pregnant mice have an impaired ability to control influenza virus infection in both lethal and non-lethal models.

### High Dose E2 Treatment Recapitulates Key Observations in Pregnancy

**Figure 2:**
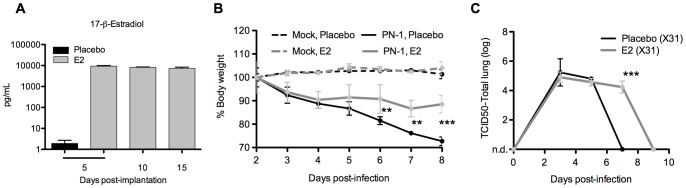
Altered influenza morbidity in estrogen-treated mice. (A) Serum levels of 17-β-estradiol were measured by competitive ELISA in E2-pelleted mice along a 15 day time course. (B) Estrogen- and placebo-pelleted mice were tracked for weight changes following infection with PN-1. (C) Total lung titers were measured by TCID50 at the indicated time points following X31 infection in pelleted mice. Non-detectable levels were indicated as n.d. Results represent mean of 3–5 mice +/− SD. ** p<0.01, ***p<0.001.

Given the well documented effects of sex hormones on immune modulation [38], [39], as well as the increased attention estradiol has received in modulating disease during pregnancy [35], [40], we decided to investigate the contribution of pregnancy-level 17-β-estradiol independently of other hormonal and immunological modulations. We made use of a biodegradable pellet that was implanted beneath the skin and release 17-β-estradiol (E2). Serum levels of 17-β-estradiol were found to be at third trimester levels [38] through the experiment (Figure 2A).

As we did with the pregnant animals, we tracked weight loss in estradiol-treated mice using a virulent PR8-derived virus (Figure 2B). Initial weight loss was nearly identical before stabilizing in E2-pelleted mice, while placebo-pelleted mice continued to lose weight throughout the latter part of infection. These observations are compatible with our results in pregnant mice, where we found more significant weight loss in non-pregnant controls.

Viral growth and clearance in E2-pelleted mice mirrored pregnancy extremely well (Figure 2C). Using X31 virus to analyze viral clearance, we found peak titers were achieved in both groups by 3dpi. Virus was cleared in the placebo-pelleted mice by 7dpi, but clearance was delayed in E2-pelleted mice. Taken together, these data support the hypothesis that E2 is at least partially responsible for the delayed viral clearance found in pregnant animals.

### Early Cytokine Dysregulation in Pregnant and E2-treated Mice

**Figure 3:**
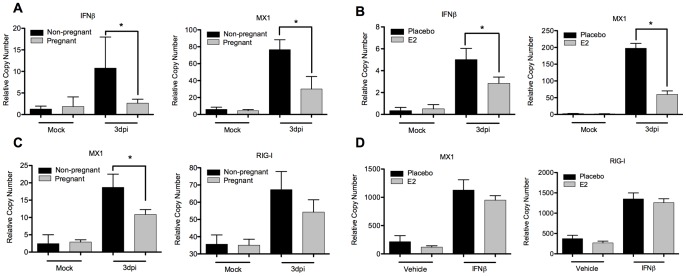
Impaired type I interferon induction in pregnant and estrogen-treated mice. (A) Lungs from X31-infected pregnant and non-pregnant mice or (B) estrogen and placebo mice were homogenized and analyzed for expression of IFNβ and the ISG MX1 by qPCR. (C) Spleens from X31-infected pregnant and non-pregnant mice were homogenized and analyzed by qPCR for the ISGs MX1 and RIG-I. (D) Splenocytes were pretreated for 18h with 10µg/mL 17-β-estradiol and directly stimulated with 1000U/mL of murine IFNβ for 6h before analysis by qPCR. Bars represent mean of 3 samples +/− SD. *p≤0.05.

One of the critical early signaling events during an influenza infection is the release of type-I interferon (IFN). We have previously demonstrated that E2 was able to significantly impair type-I interferon signaling in human dendritic cells [41]. In order to extend these observations, we measured transcription of IFNβ and interferon-stimulated genes (ISGs) by quantitative real-time PCR on RNA collected from whole lung during infection with X31. We observed a significant early impairment in this pathway in both pregnant and estrogen-treated mice (Figure 3A, 3B). The deficit was not only in IFNβ mRNA expression, but also activation of downstream ISGs, suggesting a significant, biologically relevant impairment in IFN protein expression.

We have previously shown that interferon signaling plays an important role in tissues outside the of lung [42]. Antiviral instruction of peripheral leukocytes is important for effective control of viral infection and a feature of a robust immune response. Using the spleen as a tissue that is not in direct contact with virus, we detected an impairment of ISG upregulation in the periphery (Figure 3C). This data suggests that cytokine-mediated early immune activation is inefficient in pregnant animals.

To eliminate the possibility that estrogen treatment interferes with interferon signaling pathways in addition to interferon induction, we treated splenocytes directly with murine IFN-β (Figure 3D). We found E2 to have no effect on IFN signaling.

**Figure 4:**
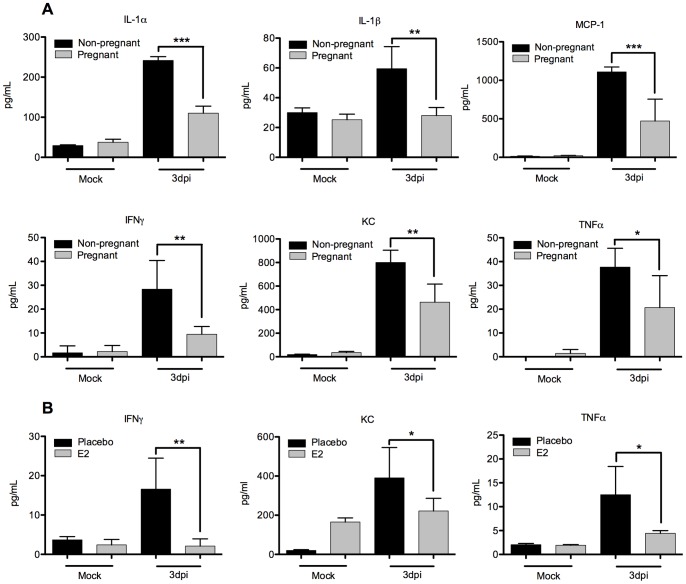
Altered cytokine signaling in pregnant and estrogen-treated mice. (A) Lung homogenates of X-31 infected pregnant and non-pregnant mice or (B) placebo and estrogen-treated mice were analyzed for cytokine production by multiplex ELISA. Bars represent mean of 3 samples +/− SD. *p≤0.05, ** p<0.01, ***p<0.001.

In order to further dissect the mechanism of altered immune responses in pregnancy, we collected whole lung digests throughout X31 infection for analysis by ELISA. At 3dpi, we observed several key early cytokines were under expressed in pregnant mice (Figure 4A), including IL-1α, IL-1β, and TNF-α. We observed reduced IFN-γ expression early in infection, as well as decreased expression of chemokines such as MCP-1 and KC. It is important to note that at this time point we do not detect any difference in viral loads.

We repeated the experiment using our estrogen model and observed a similar modulation in many of the same cytokines (Figure 4B). IFNγ, KC, and TNF-α all were under expressed in the lungs of influenza infected E2 mice compared to placebo controls. These results suggest that both models establish a significant anti-inflammatory environment at critical early time points in infection.

### Unaltered Kinetics of Innate Immune Cells

**Figure 5:**
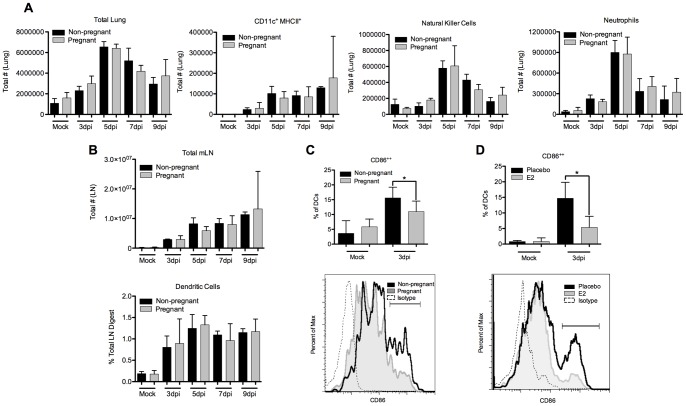
Leukocyte kinetics of migration in pregnant mice. (A) Single cell suspensions of whole lung were counted and stained for flow cytometric analysis of CD11c+ MHCII+ cells, NK1.1+ natural killer cells, and Ly6G+ neutrophils. (B) Single cell suspensions of the mediastinal LN were counted for total cells, and analyzed by flow cytometric analysis of CD11c+ MHCII+ dendritic cells. (C) CD86 expression was determined on CD11c+ DCs in the mediastinal lymph node at 3 days post infection in pregnant and (D) estrogen-treated mice. Representative histograms are shown. Bars represent mean of 3 mice +/− SD *p≤0.05.

Given the significant changes observed in early cytokine and chemokine expression, we investigated whether these changes lead to variations in migratory kinetics of early immune mediators. We did not observe any significant differences in the absolute number of cells infiltrating lung tissue during infection with X31 in pregnant (Figure 5A) or E2-treated mice (data not shown). We found no differences in populations of natural killer cells, neutrophils, or CD11c^+^ MHCII^+^ cells that represent a mixture of activated exudate macrophages, dendritic cells and infiltrated monocyte-derived cells. In order to determine whether emigration from the lung was impaired, we investigated the number of dendritic cells in the draining mediastinal lymph node at key time points of infection and found no significant differences between pregnant mice and their non-pregnant controls (Figure 5B), or between E2-pelleted mice and their placebo controls (data not shown).

We next analyzed whether the function of cells migrating into the mediastinal lymph nodes was impaired. We found strongly reduced levels of CD86 expression on CD11c+ cells at critical early time points after infection in both pregnancy (Figure 5C) and estrogen-treatment (Figure 5D), suggesting a deficiency in proper DC maturation. The maturation state of DCs at these time points is a critical junction bridging innate and adaptive immunity. The upregulation of CD86 expression, as well as other markers of activation, is extremely sensitive to IFN signaling [43]–[45].

### Impairment of CD8 T cell Function

**Figure 6:**
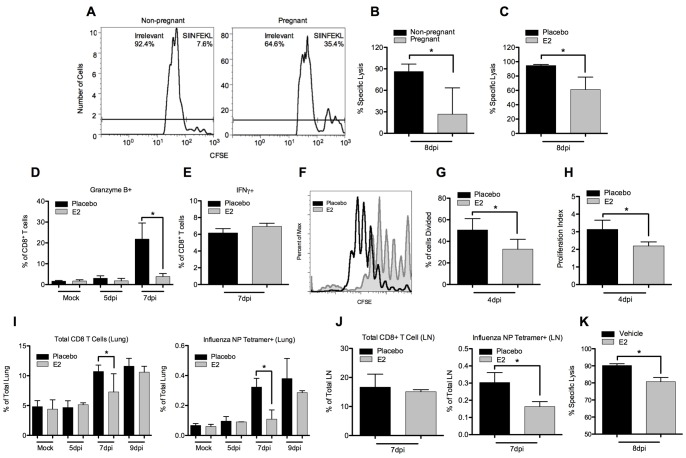
CD8 T cell functional analysis in pregnant and estrogen treated mice. (A) Representative histograms of target cells recovered from spleens of pregnant and non-pregnant mice infected with PN-1 virus. Specific lysis of antigen-bearing target cells was calculated for (B) pregnant and non-pregnant mice or (C) estrogen- and placebo-treated mice. Target cells were injected at 8dpi and analysis followed 16–18h later. (D) The percentage of CD8+ CD3+ T cells in the lung expressing high levels of Granzyme B or (E) IFN-γ were calculated in X31 infection. (F) Representative histogram overlay shows representative plots of CFSE dilution of OT-I T cells in placebo (black) and estrogen-treated (gray) mice at 4 dpi with PN-1. (G) The percentage of cells dividing and (H) proliferation index was calculated in placebo and estrogen treated mice using the proliferation platform in the FlowJo software package. (I) The percentage of total CD8+ CD3+ T cells and NP-specific tetramer+ CD8 T cells were calculated in the lung and (J) draining LN at various time points after infection with X31. (K) Specific cytotoxicity was calculated on untreated mice receiving a single 100µg dose of E2 or vehicle 6h prior to receiving target cells. Bars represent mean of 3–5 mice +/− SD *p≤0.05.

The adaptive immune response is critical for efficient clearance of viral infection. Of particular importance is the activity of cytotoxic CD8 T cells [46]. In order to determine whether functional deficiencies in adaptive immunity may account for delayed viral clearance, we made use of an in vivo cytotoxicity assay. In this assay, animals are infected with PN-1, an influenza virus bearing the model antigen SIINFEKL. Cytotoxic T cells are endogenously generated towards this peptide during infection. At 8dpi, two populations of target splenocytes are adoptively transferred. Each differentially labeled with a fluorescent dye and incubated with either SIINFEKL or an irrelevant peptide. Specific lysis of the antigen-bearing population is then calculated by comparing the relative proportion of the two target populations 16–18h after adoptive transfer (Figure 6A). We observed significant reductions in antigen-specific cytotoxicity in both pregnant (Figure 6B) and estrogen-treated (Figure 6C) animals. These differences represent delays in the ability of pregnant and E2-treated mice to clear antigen specific targets.

We next used intracellular cytokine staining techniques to determine the effect of estrogen on granzyme B production by T cells during X31 infection. Granzyme B is a serine protease that serves an important role in CD8 T cell cytotoxicity. Despite the fact that more virus is present in E2-pelleted mice at 7dpi, we found significant reductions in granzyme B production by intracellular cytokine staining (Figure 6D). In order to determine whether cytokine signaling in CD8 T cells was more broadly affected, we also measured IFN-γ production, a common measure of T cell activation. IFN-γ levels were equivalent in both placebo and estrogen treatment (Figure 6E). These results suggest that while we observe strongly reduced Granzyme B levels and impaired cytotoxicity, lung-migrating CD8 T cells were capable of secreting equivalent levels of IFNγ.

We next investigated the antigen-specific proliferative response. Animals were again infected with the PN-1 virus and adoptively transferred with CFSE-labeled OT-I T cells, which are a transgenic cytotoxic T cells specific for the SIINFEKL antigen. At 4dpi, we analyzed draining lymph nodes for CFSE dilution and found fewer CD8 T cells proliferating in estrogen treated mice (Figure 6F, 6G). Those cells that did proliferate underwent fewer divisions than their placebo-treated counterparts (Figure 6H). These results are consistent with our findings of immature dendritic cells in the lymph node at early time points.

In addition to delays in proliferation, migration of total and influenza-specific CD8 T cells to the infected lung was delayed in X31 infection (Figure 6I). These observations correlate well with the observed delays in viral clearance. Similarly, at 7dpi, antigen specific CD8 T cells in the draining lymph node continue to be reduced in estrogen-treated animals (Figure 6J). The total number of CD8 T cells is not significant reduced in the draining lymph node. This likely reflects the large number of T cells present in a lymphoid tissue.

In order to distinguish between the effects of estrogen on delayed T cell activation by antigen presenting cells and the effects independent of antigen presentation, we used a single-shot model of E2-treatment. A single 100µg dose of E2 was administered to infected mice 6h prior to adoptive transfer of target cells. This dose was sufficient to partially replicate the defect in cytotoxicity observed in E2 mice (Figure 6K). Therefore, E2 impairs T cell cytotoxicity after DC stimulation has occurred. Taken together, these data suggest two mechanisms of cytotoxic T cell deficiency. The first involving delayed proliferation and migration of antigen-specific cytotoxic T cells to the lung. The second involves a functional effect of estrogen treatment that occurs after activation of T cells and can be replicated in a short period of time after treatment.

## Discussion

Establishing a model of influenza infection during pregnancy in order to determine mechanism and intervention strategies is of primary importance. The 2009 H1N1 pandemic outbreak re-established the risk of severe morbidity and increased mortality that influenza exposure presents to pregnant women, especially in the second and third trimesters [18]–[20]. Significantly elevated mortality has been reported in mouse models [28]–[31]. Using an aerosol model of infection, we were able to detect quantifiable and clinically relevant increases in morbidity independent of lethality. Notably, fetal pups were found to be significantly underweight, and were delivered prematurely, consistent with clinical observations [23], [37]. In addition to these measures of morbidity, we also observed evidence of an anti-inflammatory phenotype in pregnant mice. Viral clearance was delayed in pregnancy, using both lethal and non-lethal infections. Additionally, weight loss in pregnant mice was attenuated and delayed.

Altered pathology in response to influenza virus infection during pregnancy is likely mediated by multiple factors including changes to sex hormones and increased cardiopulmonary demands [47]. We focused on the impact of pregnancy-levels of estrogen for its well-defined impacts on immune function. Estrogen is a particularly interesting immune modulator as it has been described as having bimodal effects on immunity [38]. Circulating levels of estrogen in female mice have been associated with stimulating immune function in general, including exacerbating influenza virus pathogenesis [48]. This accounts for the relative susceptibility to influenza virus infection in non-pregnant females compared to males. This effect can be abolished by gonadectomy. Gonadally-intact females were used in this study in order to directly compare pathogenesis in pregnancy or high-dose estrogen-treatment to healthy wild-type females.

High levels of estrogen, on the other hand, have been associated with an anti-inflammatory phenotype, including in the context of influenza A virus infection [48], and cannot be directly linked to the susceptibility of pregnant females to high morbidity. High-dose estrogen treatment has been used to alleviate symptoms in models of rheumatoid arthritis [34], [49] and multiple sclerosis [32], [33], [50] in response to clinical observations showing a reduction of symptoms in pregnant women with these diseases.

Excessive immune responses mediate much of the pathology associated with influenza virus [51], [52]. Pregnancy-specific morbidities have also been associated with inflammation including premature parturition [53]–[55]. Anti-inflammatory treatment can prevent loss of pregnancy. Blocking TNF-α or administering anti-inflammatory cytokines such as IL-10 [56] have been shown to reduce morbidity. Additionally, the placenta has been shown to employ negative regulatory mechanisms aimed at reducing inflammatory cytokine signaling [57]. Given the well described anti-inflammatory phenotype of estrogen, and specifically the high levels of estrogen associated with pregnancy, we sought to investigate what role estrogen can play in the context of increased morbidity to influenza infection during pregnancy.

While other groups have reported on increased morbidity to influenza infection during pregnancy, anti-inflammatory mechanisms in the context of infection have not been extensively characterized as they have been in the context of disease [6], [7] and of fetal rejection [3], [5]. Because of a focus on morbidity, mouse models of influenza virus infection during pregnancy often rely on a high dose intranasal inoculum of pathogenic virus strains such as the 2009 Pandemic H1N1 strain. Using these models, morbidities such as elevated mortality and low fetal birth weight have been observed [30], [31]. Clinical observations of preterm delivery have not been reported in a mouse model, and was not observed in one study [30], despite increased cytokines levels. Additionally, pro-inflammatory cytokine phenotypes have been reported in these models, and delays in viral clearance are often not supported, although these reports often focus on events late in infection or reflect divergent viral kinetics.

We were concerned that models using highly pathogenic intranasal inoculation may not adequately model clinical presentations of influenza virus infection and obscure relevant phenotypes. Aerosolized infection closely models clinical disease [58], [59] and was able to replicate clinical observations of influenza infection during pregnancy. We focused on a early cytokine events within the first 24h of the onset of immunity in our model [36]. These inflammatory events have broad impacts on the outcome of immunity, and serve as the best indicators early inflammation.

By making use of a model of infection using pregnancy-levels of 17-β-estradiol, we were able to measure its impact on immunity independently of other pregnancy-associated variables. In addition to demonstrating a similar delay in viral clearance, estrogen-treated animals were also protected from the weight loss experienced by placebo controls. It is not clear how estrogen may protect mice from weight loss associated with influenza infection, but a likely mechanism may be decreased cytokine-associated cachexia.

In both pregnant and estrogen-treated animals, early cytokine responses were reduced. Estrogens have been linked to modulation of cytokine signaling, such as through antagonism of NF-κB pathway [38], [60]. Sources often disagree as to the degree and direction of these changes depending on the concentration of estrogen, model organism, and method of stimulation [38], [39], [61]. Indeed, we saw a strong trend towards elevated levels of the chemokine KC in the steady state of pregnancy, followed by significant deficit in KC expression upon infection. This complex regulation highlights the elaborate mechanisms involved in estrogen signaling [62]. Pregnancy presents additional layers of complexity in cytokine regulation. Several hormones circulating in pregnancy are immunologically active, including progesterone and prolactin [39], [63]. We did not observe identical regulation of all cytokines in pregnancy and estrogen-treatment, and some of these other hormones may account for some of this variation. The overlap remains significant, and both models demonstrate defects in early immune responses.

Over-expression of several regulated cytokines including IL-1α, IL-1β, TNF-α, and IFN-γ have been associated with morbidities in influenza infection [52], and spontaneous pregnancy loss [56], [64]. The ability of estrogen to suppress cytokine expression has been proposed as protective in influenza virus pathogenesis [48]. Indeed, it may be that estrogen plays a protective role during pregnancy by muting early cytokine responses that would otherwise threaten fetal development [22], [54]. Enhanced morbidities experienced during pregnancy may rely on the failure to clear virus following an impaired immune response. Prolonged infection may result in a late cytokine response triggering premature delivery and significant morbidity. Delayed weight loss in pregnant animals supports the hypothesis of a late development inducing pathology. Alternatively, prolonged infection may lead to significant lung damage. Others have reported that delayed lung repair during pregnancy leads significant cardiopulmonary demands during pregnancy, and may contribute to morbidity [31].

Type-I IFNs play a critical role in early immune responses to influenza, and impairment of this pathway during pregnancy implicates a significant alteration in the response to virus. Transcription of IFN-β is a prototypical marker of early type-I interferon expression, and was found to be significantly reduced in both of our models. We did not find any evidence that estrogen treatment interfered with downstream signaling pathways, suggesting that this effect is mediated by reduced type-I interferon expression. ISG levels were reduced not only in the lung, but additionally in the periphery. This is an important observation, as interferon signaling in the periphery is necessary for efficient antiviral immunity [42]. This reduction is well conserved in human models of both E2 treatment and pregnancy [13], [41]. The mechanism of interaction between estrogen and type-I IFN induction pathways is not currently understood, but could lead to significant insights.

After the onset of inflammation, pregnant and E2-treated animals were able to control early virus titers as well as wild-type counterparts, despite reduced levels of interferon. Influenza virus NS1 is a powerful inhibitor of inflammation and functions to counteract early checks on viral growth [36], likely minimizing the impact of interferon deficiency at the earliest time points. Interferon signaling also plays an important role in the activation and maturation of antigen-bearing dendritic cells [65], [66], and serves as a bridge between innate and adaptive immunity [44], [45]. Pregnancy-levels of estrogen were able to interfere with proper dendritic cell maturation. The reduced levels of the co-stimulatory molecule CD86 is particularly relevant for its role in CD8 T cell activation [67].

The primary goal of dendritic cell maturation is the efficient initiation of adaptive immunity, and in particular initiating proliferation of antigen-specific CD8 T cells [46]. As expected, we observed significant estrogen-associated delays in CD8 T cell proliferation in the lymph node at 4dpi, and significantly reduced numbers of antigen-specific cytotoxic T cells in the lung and lymph node at 7dpi. These numbers eventually recovered by 9dpi, correlating nicely with the kinetics of viral clearance in this model. Because the observed phenotype results in delays in CD8 T cell recruitment to the lung, the timing of this observation is critical. Observations made late in infection may not reflect this phenotype and may explain why others have failed to note significant differences in the numbers of cytotoxic T cells in similar models [31]. Dysregulation of interferon function can be linked to delays in appropriate activation of cytotoxic CD8 T cells, and delays in viral clearance [68].

In addition to experiencing delays in proliferation and migration, these T cells are functionally compromised. Cytotoxicity was decreased in an in vivo cytotoxicity assay, and granzyme B levels were strongly reduced in the lungs of infected mice. Interestingly, IFN-γ expression in CD8 T cells was unchanged, suggesting that these cells that reach the lung were at least partially activated, even while functionally compromised. A single dose of estrogen administered immediately prior to encountering target cells was sufficient to impair cytotoxic function, suggesting that this effect is not entirely mediated by inefficient dendritic cell stimulation. Estrogen signaling pathways have been associated with lysosome biogenesis in cytotoxic T cells, and may represent a relevant mechanism [69].

Notably, two mechanisms of impaired adaptive immunity can be observed. The first involves delayed proliferation in the draining lymph nodes and can account for delayed migration to the lung. This mechanism is likely the product of weak early interferon signaling events and impaired antigen presentation. Those cells that do proliferate and are activated are additionally functionally compromised. This second mechanism is independent of antigen presentation and is at least partially responsible for a deficiencies in cytotoxic function. This mechanism has a rapid onset and can be observed within hours of estrogen treatment. This mechanism likely accounts for the observed decreases in granzyme B production that do not effect IFN-γ levels in the same T cells.

Although pregnancy is a complex immunological state, it is clear that estrogen plays a key role. Initially, estrogen reduces early cytokine responses and may function to protect from cytokine-associated morbidities. Early recruitment of leukocytes is equivalent and early viral titers are controlled equally well in pregnant and wild type mice. The key deficit appears to be in priming of dendritic cells that migrate from the lungs to lymph nodes to initiate T cell expansion. This disruption in interferon signaling leads to suboptimal CD8 T cell activation and delayed viral clearance. Estrogen also appears to impair CD8 T cell cytotoxicity independently of dendritic cell activity. It is not currently clear how these anti-inflammatory effects impact the viability of pregnancy, particularly fetal development and premature delivery, but evidence suggests it may play a protective role. Deficits in viral clearance and prolonged exposure to infection may be act as stressors that trigger delayed morbidities despite the anti-inflammatory effects of estrogen. It is also not clear whether prolonged infection is of significant impact on morbidity absent pregnancy, as would be the case in hormone treatment. These are key questions that require further study and the establishment of novel models.

There is overwhelming clinical value to understanding the mechanisms and contributions of sex hormones to immune modulation during pregnancy. Animal models, such as those described in this report, allow for controlled infection conditions and detailed observation of early events during infection. Understanding the mechanisms and immunological outcomes of pregnancy, particularly the role of steroid hormones, may lead to the development of therapeutic options for protection of women, their children, and patients receiving hormone replacement therapy. Establishing the tenets of hormone mediated immune modulation may provide testable hypotheses for treatment of autoimmune diseases such as multiple sclerosis.

## Materials and Methods

### Ethics Statement

All animal work was conducted in agreement with approved protocols by the Institutional Animal Care and Use Committee (IACUC) at the Mount Sinai School of Medicine (Protocol Number #: 09-1313) and in accordance with guidelines in the Guide for the Care and Use of Laboratory Animals of the National Institutes of Health. The program is fully accredited by the Association for Assessment & Accreditation of Laboratory Animal Care, International (AAALAC).

### Mice

C57BL/6 wild type, transgenic OT-I and syngeneic pregnant mice were purchased from Jackson Laboratories (Bar Harbor, MA). Timed pregnant mice were delivered at E8.5 and allowed two days to rest before commencing the experiment. Non-pregnant mice were age matched to timed pregnant mice. Wild-type mice were implanted with placebo or estrogen pellets purchased from Innovative Research of America. Estrogen pellets contained 35mg of 17-β-estradiol and were designed for 21-day release. Surgery was performed 4–5 days prior to infection. Mice were anesthetized with Avertin (Tribromoethanol, Acros Organics) and shaved behind the right ear. The pellet was implanted via small incision between the shoulder and ear and secured with a surgical clip. Mice were monitored for signs of infection. For experiments using a single dose of estradiol, mice received a single 100µg subcutaneous injection of water soluble 17-β-estradiol (Sigma) six hours prior to receiving target cells for in vivo cytotoxicity assay. The animals were housed in specific pathogen-free conditions.

### Viruses and Infection

Influenza virus strains A/PR/8/1934 (H1N1) (PR8), recombinant PR8-OTI (H1N1) (PN-1), and A/X-31(H3N2) (X31) were propagated in 10-day-old embryonated eggs. Virus titers were determined by tissue culture infectivity assay (TCID50) as previously described [70]. Briefly, lungs were extracted and homogenized in PBS-gelatin (0.1%) and frozen in dry ice-ethanol for storage at −80°C. Presence of the virus was detected by infecting MDCK cells at 1∶10 dilutions for 72h in the presence of trypsin. Presence of virus was measure by testing for the presence of hemagglutination of chicken red blood cells. The limit of detection for this assay is approximately 8×10^2^ TCID50 per lung.

All mice were infected using an Inhalation Exposure System A42X (Glass-Col, USA). Virus was diluted in PBS to obtain a solution of 10^7.9^ TCID50 in12mL. This solution was placed in a glass nebulizer and aerosolized for a total exposure of 30 min. This leads to 100% infection rate as described previously [36], [71]. We estimate between 10 and 100 infectious particles are passively inhaled during this period [72]. Mock infected animals are exposed to aerosolized PBS.

### Real-time quantitative PCR (qPCR)

Lungs or spleen at indicated time points were homogenized in 3mL of Trizol Reagent (Invitrogen). RNA was isolated as indicated by the manufacturer, and converted to cDNA by RT-PCR using the Transcriptor First Strand cDNA Synthesis system (Roche). qPCR reactions were performed using a Lightcycler 480 II (Roche). All reactions were normalized to three housekeeping genes: α-Tubulin, RPS-11, and β-actin, as previously described [73]. The following primers were used in this study: β-Actin – Forward: AGGTGACAGCATTGCTTCTG, Reverse: GCTGCCTCAACACCTCAAC; α-Tubulin – Forward: TGCCTTTGTGCACTGGTATG, Reverse: CTGGAGCAGTTTGACGACAC; RPS-11 – Forward: CGTGACGAAGATGAAGATGC, Reverse: GCACATTGAATCGCACAGTC; IFNβ – Forward: AGATGTCCTCAACTGCTCTC, Reverse: AGATTCACTACCAGTCCCAG; MX1 – Forward: CAACTGGAATCCTCCTGGAA, Reverse: GGCTCTCCTCAGAGGTATCA; RIG-I – Forward: CAGACAGATCCGAGACACTA, Reverse: TGCAAGACCTTTGGCCAGTT.

### Flow Cytometry

Lungs were perfused with cold PBS containing 0.5mM EDTA and immediately ground in Hank's Buffered Saline Solution containing 0.5mM EDTA and 0.5% FBS using the gentleMACS Tissue Dissociator (Miltenyi Biotec). Mediastinal lymph nodes were mechanically disrupted. Both tissues were then incubated with 0.25mg/mL collagenase (Liberase type III, Roche) and 8000U/mL DNAse I (Invitrogen) for 20 min at 37°C. Collagenase was inactivated with sterile HBSS containing 2% FBS and the suspension was passed through a 70µm strainer. Suspensions were then treated with red blood cell lysis buffer (BD Biosciences), and resuspended in HBSS containing 10µg/mL Fc-receptor Block (BD Biosciences). Total cell counts were then attained by hemocytometer. Cells were then stained with antibodies for multiple surface antigens: CD8 (53–6.7), CD3 (17A2), CD11c (HL3), MHCII (M5/114.15.2), CD86 (GL-1), NK1.1 (PK136), Ly6G (1A8). For intracellular cytokine staining, total lung was digested and a single cell suspension was incubated with Brefeldin A-containing GolgiPlug (BD Biosciences) for six hours, according to manufacturer's instructions. Following staining of extracellular antigens, intracellular cytokine staining was performed using the Cytofix/Cytoperm system (BD Biosciences). Intracellular antigens Granzyme B (GB11), and IFN-γ (XMG1.2) were stained following permeabilization. Antibodies were purchased from BD Bioscience, eBiosciences, and Biolegend. Kb/SIINFEKL pentamer was purchased from ProImmune. Influenza NP-specific tetramers were kindly provided by Dr. David Woodland (Trudeau Institute). Samples were acquired using Cytomic FC500 Coulter Station (Beckman Coulter) and analyzed using Flow Jo software (Treestar Corp).

### ELISA

Whole lung was homogenized in PBS-gelatin (0.1%). Cytokine concentration was measured by bead-based multiplex ELISA (Millipore) using a Luminex 200 (Luminex Corporation). Serum was used for detection of circulating 17-β-estradiol using a competitive estradiol ELISA (Cayman Chemicals).

### In vivo Cytotoxicity

Mice were infected as described with PN-1 virus or mock infected with PBS. At 8dpi, 1–2.5×10^6^ target cells were adoptively transferred. Splenocytes from a congenic naive donor mouse were used as target cells, and injected intravenously as a 1∶1 ratio of two populations. One population was incubated with 100µg/mL of the H2-K^b^-restricted OVA peptide, SIINFEKL, and labeled with 2.5µM CFSE. The second population was incubated with irrelevant peptide and labeled with 0.25µM CFSE. 16–18h post-transfer mice were sacrificed. Spleens of recipient mice were disrupted, and suspended in red blood cell lysis buffer. Total spleen was analyzed by flow cytometry for the relative proportion of the CFSE-labeled cells. Specific killing was calculated as previously described [74]. Briefly, a normalization value (N) was calculated as (% Low CFSE/% High CFSE) in mock infected animals for each treatment group. Percent specific cytotoxicity was then calculated for each infected mouse as follows: (% Low CFSE x N – % High CFSE)/(% Low CFSE x N) X 100.

### T cell Isolation and CFSE Dilution

Spleens from OT-I mice were mechanically disrupted and passed through a 70µm strainer. Cells were suspended in red blood cell lysis buffer (BD Biosciences), washed and suspended in HBSS containing 0.5% FBS and 10µg/mL Fc-receptor Block (BD Biosciences). Cells were then incubated with a cocktail of biotin-labeled antibodies including: CD19 (1D3), B220 (RA3-6B2), MHCII (M5/114.15.2), GR-1 (RB6-8C5), NK1.1 (PK136), CD11b (M1/70), Ter119 (TER119), and CD4 (GK1.5). Cell suspensions were then washed and incubated with Anti-biotin magnetic beads and passed over a MACS magnetic column (Miltenyi Biotec). Flow through was collected and verified by flow cytometry to have a purity of greater than 80%. For the CFSE dilution assay, these cells were stained with 2.5µM CFSE. 1×10^6^–2.5×10^6^ labeled CD8 T cells were intravenously injected into recipient mice on the day of infection. At 4dpi, mice were sacrificed and lymph nodes were collected for analysis.

### Statistical Analysis

Results are expressed as mean +/− standard deviation. Data sets with multiple groups were analyzed by 1-way ANOVA followed by Newman-Keuls multiple comparison test. Weight loss was evaluated using a two-way ANOVA. Single time point analyses were evaluated using unpaired two-tailed Student's t test. P values ≤ 0.05 (95% Confidence) were considered to be significant. Data was analyzed and graphs were prepared using Prism 5 software.
